# Deacetyl­ tenuazonic acid

**DOI:** 10.1107/S1600536809015372

**Published:** 2009-05-07

**Authors:** David Siegel, Matthias Koch, Franziska Emmerling, Irene Nehls

**Affiliations:** a Bundesanstalt für Materialforschung und -prüfung, Abteilung Analytische Chemie; Referenzmaterialien, Richard-Willstätter-Strasse 11, D-12489 Berlin-Adlershof, Germany

## Abstract

The heterocycle in the title compound {systematic name: (5*S*)-5-[(1*S*)-1-methyl­prop­yl]pyrrolidine-2,4-dione}, C_8_H_13_NO_2_, is planar (r.m.s. deviation for all non-H atoms = 0.008 Å). The crystal structure is stabilized by N—H⋯O hydrogen bonding.

## Related literature

Tenuazonic acid (TA) is an *Alternaria* mycotoxin commonly encountered in food (Siegel, Rasenko *et al.*, 2009 ▶; Weidenbörner, 2001 ▶). The title compound is known to be formed upon boiling TA in 0.1 *M* HCl (Stickings, 1959 ▶). For the synthesis of the title compound, see: Lebrun *et al.* (1988 ▶). For the crystal structure of the tenuazonic acid copper (II) salt, see: Dippenaar *et al.* (1977 ▶) and for the 2,4-dinitro­phenyl­hydrazone, see: Siegel, Merkel *et al.* (2009 ▶). For the structures of other pyrrolidine-2,4-diones, see, for example: Yu *et al.* (2007 ▶); Zhu *et al.* (2004 ▶); Ellis & Spek (2001 ▶).
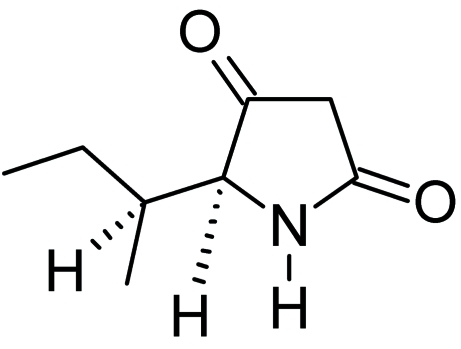


## Experimental

### 

#### Crystal data

C_8_H_13_NO_2_
*M* *_r_* = 155.19
Monoclinic, 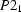

*a* = 5.0114 (4) Å
*b* = 7.7961 (4) Å
*c* = 10.9919 (10) Å
β = 95.778 (4)°
*V* = 427.26 (6) Å^3^
*Z* = 2
Cu *K*α radiation
μ = 0.71 mm^−1^
*T* = 193 K
0.44 × 0.16 × 0.16 mm

#### Data collection

Enraf–Nonius CAD-4 diffractometer
Absorption correction: ψ scan (*CORINC*; Dräger & Gattow, 1971 ▶) *T*_min_ = 0.744, *T*_max_ = 0.993 (expected range = 0.669–0.893)
1866 measured reflections
1571 independent reflections
1558 reflections with *I* > 2σ(*I*)
*R*_int_ = 0.040
3 standard reflections frequency: 60 min intensity decay: 2%

#### Refinement

*R*[*F* ^2^ > 2σ(*F* ^2^)] = 0.036
*wR*(*F* ^2^) = 0.098
*S* = 1.06
1571 reflections
103 parameters
1 restraint
H-atom parameters constrained
Δρ_max_ = 0.22 e Å^−3^
Δρ_min_ = −0.17 e Å^−3^
Absolute structure: Flack (1983 ▶), 697 Friedel pairs
Flack parameter: 0.1 (2)

### 

Data collection: *CAD*-4 *Software* (Enraf–Nonius, 1989 ▶); cell refinement: *CAD*-4 *Software*; data reduction: *CORINC* (Dräger & Gattow, 1971 ▶); program(s) used to solve structure: *SIR97* (Altomare *et al.*, 1999 ▶); program(s) used to refine structure: *SHELXL97* (Sheldrick, 2008 ▶); molecular graphics: *ORTEPIII* (Burnett & Johnson, 1996 ▶); software used to prepare material for publication: *SHELXTL* (Sheldrick, 2008 ▶).

## Supplementary Material

Crystal structure: contains datablocks I, global. DOI: 10.1107/S1600536809015372/bt2937sup1.cif

Structure factors: contains datablocks I. DOI: 10.1107/S1600536809015372/bt2937Isup2.hkl

Additional supplementary materials:  crystallographic information; 3D view; checkCIF report

## Figures and Tables

**Table 1:** Hydrogen-bond geometry (Å, °)

*D*—H⋯*A*	*D*—H	H⋯*A*	*D*⋯*A*	*D*—H⋯*A*
N1—H1⋯O1^i^	0.90	2.02	2.8963 (18)	164

Symmetry code: (i) 
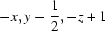
.

**Fig. 1.**
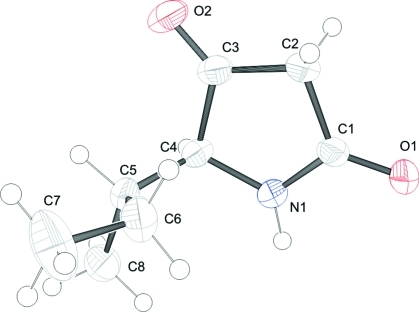
ORTEP representation of the title compound with atomic labeling of, shown with 50% probability displacement ellipsoids.

**Fig. 2.**
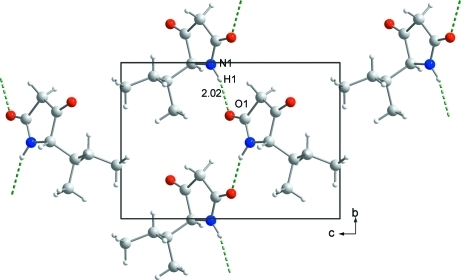
View of the unit cell of the title compound along [100], showing the hydrogen-bonded chains running along the twofold screw axis.

## supplementary crystallographic information

### Comment

Tenuazonic acid (TA) is an *Alternaria* mycotoxin commonly encountered in food (Siegel, Rasenko *et al.*, 2009; Weidenbörner, 2001). The title compound is known to be formed upon boiling of TA in 0.1 *M* HCl (Stickings, 1959). It is therefore a possible degradation product which might also be encountered in food matrices.

Whereas TA itself could so far only be crystallized as its copper (II) salt (Dippenaar *et al.*, 1977) or 2,4-dinitrophenylhydrazone (Siegel, Merkel *et al.*, 2009), the title compound is conveniently crystallized from hexane/ethyl acetate.

Each molecule (Fig. 1) is connected to two adjacent molecules *via* N—H···O hydrogen bonds. Along the *b* axis chains of symmetry equivalent molecules are formed (Fig. 2).

### Experimental

The title compound was supplied by the workgroup of Professor *R*. Faust (University of Kassel, Germany) by synthesis according to a literature procedure (Lebrun *et al.*, 1988). For *x*-ray analysis, it was recrystallized several times from hexane:ethyl acetate 50:50 (v:v).

### Refinement

The hydrogen atoms were located in difference maps but positioned with idealized geometry and refined using the riding model, with C—H = 0.98–1.00 Å or N—H = 0.90 Å and *U*_iso_(H) = 1.2*U*_eq_(C,N) or 1.5*U*_eq_(C_methyl_).

### Crystal data

**Table:**

C_8_H_13_NO_2_	*F*(000) = 168
*M**_r_* = 155.19	*D*_x_ = 1.206 Mg m^−^^3^
Monoclinic, *P*2_1_	Cu *K*α radiation, λ = 1.54178 Å
Hall symbol: P 2yb	Cell parameters from 25 reflections
*a* = 5.0114 (4) Å	θ = 67–69°
*b* = 7.7961 (4) Å	µ = 0.71 mm^−^^1^
*c* = 10.9919 (10) Å	*T* = 193 K
β = 95.778 (4)°	Block, yellow
*V* = 427.26 (6) Å^3^	0.44 × 0.16 × 0.16 mm
*Z* = 2	

### Data collection

**Table:**

Enraf–Nonius CAD-4 diffractometer	1558 reflections with *I* > 2σ(*I*)
Radiation source: rotating anode	*R*_int_ = 0.040
graphite	θ_max_ = 69.9°, θ_min_ = 4.0°
ω/2θ scans	*h* = −6→5
Absorption correction: ψ scan (CORINC; Dräger & Gattow, 1971)	*k* = −8→9
*T*_min_ = 0.744, *T*_max_ = 0.993	*l* = −13→13
1866 measured reflections	3 standard reflections every 60 min
1571 independent reflections	intensity decay: 2%

### Refinement

**Table:**

Refinement on *F*^2^	Hydrogen site location: inferred from neighbouring sites
Least-squares matrix: full	H-atom parameters constrained
*R*[*F*^2^ > 2σ(*F*^2^)] = 0.036	*w* = 1/[σ^2^(*F*_o_^2^) + (0.0616*P*)^2^ + 0.0771*P*] where *P* = (*F*_o_^2^ + 2*F*_c_^2^)/3
*wR*(*F*^2^) = 0.098	(Δ/σ)_max_ < 0.001
*S* = 1.06	Δρ_max_ = 0.22 e Å^−^^3^
1571 reflections	Δρ_min_ = −0.16 e Å^−^^3^
103 parameters	Extinction correction: *SHELXL97* (Sheldrick, 2008), Fc^*^=kFc[1+0.001xFc^2^λ^3^/sin(2θ)]^-1/4^
1 restraint	Extinction coefficient: 0.017 (4)
Primary atom site location: structure-invariant direct methods	Absolute structure: Flack (1983), 697 Friedel pairs
Secondary atom site location: difference Fourier map	Flack parameter: 0.1 (2)

### Special details

**Table:**

Geometry. All e.s.d.'s (except the e.s.d. in the dihedral angle between two l.s. planes) are estimated using the full covariance matrix. The cell e.s.d.'s are taken into account individually in the estimation of e.s.d.'s in distances, angles and torsion angles; correlations between e.s.d.'s in cell parameters are only used when they are defined by crystal symmetry. An approximate (isotropic) treatment of cell e.s.d.'s is used for estimating e.s.d.'s involving l.s. planes.
Refinement. Refinement of *F*^2^ against ALL reflections. The weighted *R*-factor *wR* and goodness of fit *S* are based on *F*^2^, conventional *R*-factors *R* are based on *F*, with *F* set to zero for negative *F*^2^. The threshold expression of *F*^2^ > σ(*F*^2^) is used only for calculating *R*-factors(gt) *etc*. and is not relevant to the choice of reflections for refinement. *R*-factors based on *F*^2^ are statistically about twice as large as those based on *F*, and *R*- factors based on ALL data will be even larger.

### Fractional atomic coordinates and isotropic or equivalent isotropic displacement parameters (Å^2^)

**Table:**

	*x*	*y*	*z*	*U*_iso_*/*U*_eq_	
O1	−0.0559 (2)	0.66044 (16)	0.50335 (11)	0.0386 (3)	
O2	0.5911 (3)	0.74694 (19)	0.22570 (14)	0.0494 (4)	
N1	0.2318 (3)	0.48864 (18)	0.41048 (11)	0.0303 (3)	
H1	0.1866	0.3946	0.4519	0.036*	
C1	0.1196 (3)	0.6392 (2)	0.43372 (14)	0.0305 (3)	
C2	0.2400 (3)	0.7779 (2)	0.36022 (15)	0.0360 (4)	
H2A	0.1012	0.8320	0.3021	0.043*	
H2B	0.3265	0.8675	0.4145	0.043*	
C3	0.4437 (3)	0.6853 (2)	0.29328 (15)	0.0335 (4)	
C4	0.4354 (3)	0.4941 (2)	0.32393 (13)	0.0296 (3)	
H4	0.6122	0.4594	0.3672	0.036*	
C5	0.3757 (3)	0.3822 (2)	0.20975 (14)	0.0317 (4)	
H5	0.5040	0.4161	0.1500	0.038*	
C6	0.0924 (4)	0.4130 (3)	0.14885 (16)	0.0425 (4)	
H6A	−0.0373	0.3594	0.1995	0.051*	
H6B	0.0571	0.5380	0.1467	0.051*	
C7	0.0441 (6)	0.3429 (4)	0.0202 (2)	0.0761 (8)	
H7A	0.1721	0.3946	−0.0307	0.114*	
H7B	−0.1391	0.3706	−0.0138	0.114*	
H7C	0.0679	0.2181	0.0219	0.114*	
C8	0.4266 (4)	0.1937 (2)	0.2416 (2)	0.0474 (5)	
H8A	0.6088	0.1804	0.2821	0.071*	
H8B	0.4071	0.1250	0.1666	0.071*	
H8C	0.2967	0.1549	0.2966	0.071*	

### Atomic displacement parameters (Å^2^)

**Table:**

	*U*^11^	*U*^22^	*U*^33^	*U*^12^	*U*^13^	*U*^23^
O1	0.0498 (7)	0.0316 (6)	0.0377 (6)	−0.0010 (5)	0.0201 (5)	−0.0054 (5)
O2	0.0530 (8)	0.0418 (8)	0.0576 (8)	−0.0067 (6)	0.0262 (6)	0.0115 (6)
N1	0.0349 (7)	0.0268 (7)	0.0303 (6)	−0.0028 (5)	0.0094 (5)	0.0017 (5)
C1	0.0374 (8)	0.0264 (8)	0.0279 (7)	−0.0056 (6)	0.0046 (6)	−0.0030 (6)
C2	0.0462 (9)	0.0253 (8)	0.0378 (8)	−0.0062 (7)	0.0104 (7)	−0.0022 (7)
C3	0.0348 (8)	0.0312 (8)	0.0347 (8)	−0.0065 (6)	0.0043 (6)	0.0024 (7)
C4	0.0268 (7)	0.0308 (8)	0.0321 (7)	−0.0025 (6)	0.0063 (5)	0.0042 (7)
C5	0.0310 (8)	0.0312 (8)	0.0346 (8)	0.0006 (6)	0.0120 (6)	−0.0011 (6)
C6	0.0373 (9)	0.0512 (11)	0.0391 (9)	0.0028 (7)	0.0037 (7)	−0.0102 (8)
C7	0.0824 (18)	0.087 (2)	0.0547 (14)	0.0218 (14)	−0.0144 (12)	−0.0302 (13)
C8	0.0558 (11)	0.0317 (9)	0.0573 (12)	0.0060 (8)	0.0182 (8)	−0.0003 (8)

### Geometric parameters (Å, °)

**Table:**

O1—C1	1.2338 (19)	C5—C8	1.526 (2)
O2—C3	1.199 (2)	C5—C6	1.526 (2)
N1—C1	1.337 (2)	C5—H5	1.0000
N1—C4	1.4640 (18)	C6—C7	1.512 (3)
N1—H1	0.9038	C6—H6A	0.9900
C1—C2	1.511 (2)	C6—H6B	0.9900
C2—C3	1.501 (2)	C7—H7A	0.9800
C2—H2A	0.9900	C7—H7B	0.9800
C2—H2B	0.9900	C7—H7C	0.9800
C3—C4	1.530 (2)	C8—H8A	0.9800
C4—C5	1.533 (2)	C8—H8B	0.9800
C4—H4	1.0000	C8—H8C	0.9800
			
C1—N1—C4	115.63 (14)	C6—C5—C4	111.45 (13)
C1—N1—H1	119.0	C8—C5—H5	107.6
C4—N1—H1	125.2	C6—C5—H5	107.6
O1—C1—N1	125.18 (14)	C4—C5—H5	107.6
O1—C1—C2	125.70 (14)	C7—C6—C5	114.04 (16)
N1—C1—C2	109.12 (14)	C7—C6—H6A	108.7
C3—C2—C1	104.25 (14)	C5—C6—H6A	108.7
C3—C2—H2A	110.9	C7—C6—H6B	108.7
C1—C2—H2A	110.9	C5—C6—H6B	108.7
C3—C2—H2B	110.9	H6A—C6—H6B	107.6
C1—C2—H2B	110.9	C6—C7—H7A	109.5
H2A—C2—H2B	108.9	C6—C7—H7B	109.5
O2—C3—C2	127.06 (17)	H7A—C7—H7B	109.5
O2—C3—C4	123.96 (16)	C6—C7—H7C	109.5
C2—C3—C4	108.98 (13)	H7A—C7—H7C	109.5
N1—C4—C3	101.98 (13)	H7B—C7—H7C	109.5
N1—C4—C5	115.10 (13)	C5—C8—H8A	109.5
C3—C4—C5	112.44 (13)	C5—C8—H8B	109.5
N1—C4—H4	109.0	H8A—C8—H8B	109.5
C3—C4—H4	109.0	C5—C8—H8C	109.5
C5—C4—H4	109.0	H8A—C8—H8C	109.5
C8—C5—C6	112.26 (15)	H8B—C8—H8C	109.5
C8—C5—C4	110.21 (14)		
			
C4—N1—C1—O1	179.64 (15)	C2—C3—C4—N1	−1.75 (16)
C4—N1—C1—C2	0.22 (18)	O2—C3—C4—C5	−57.6 (2)
O1—C1—C2—C3	179.26 (14)	C2—C3—C4—C5	122.08 (14)
N1—C1—C2—C3	−1.33 (18)	N1—C4—C5—C8	−75.53 (17)
C1—C2—C3—O2	−178.44 (17)	C3—C4—C5—C8	168.27 (14)
C1—C2—C3—C4	1.90 (17)	N1—C4—C5—C6	49.81 (19)
C1—N1—C4—C3	0.95 (16)	C3—C4—C5—C6	−66.39 (17)
C1—N1—C4—C5	−121.07 (15)	C8—C5—C6—C7	−70.6 (3)
O2—C3—C4—N1	178.57 (16)	C4—C5—C6—C7	165.2 (2)

### Hydrogen-bond geometry (Å, °)

**Table:**

*D*—H···*A*	*D*—H	H···*A*	*D*···*A*	*D*—H···*A*
N1—H1···O1^i^	0.90	2.02	2.8963 (18)	164

Symmetry codes: (i) −*x*, *y*−1/2, −*z*+1.
